# Changes in the Genetic Structure of Lithuania’s Wild Boar (*Sus scrofa*) Population Following the Outbreak of African Swine Fever

**DOI:** 10.3390/genes13091561

**Published:** 2022-08-30

**Authors:** Loreta Griciuvienė, Žygimantas Janeliūnas, Simona Pilevičienė, Vaclovas Jurgelevičius, Algimantas Paulauskas

**Affiliations:** 1 Faculty of Natural Sciences, Vytautas Magnus University, K. Donelaičio 58, 44248 Kaunas, Lithuania; 2 National Food and Veterinary Risk Assessment Institute, J. Kairiūkščio 10, 08409 Vilnius, Lithuania

**Keywords:** wild boar, genetic structure, African swine fever, Lithuania

## Abstract

The emergence of African swine fever (ASF) in Lithuania and its subsequent persistence has led to a decline in the population of wild boar (*Sus scrofa*). ASF has been spreading in Lithuania since its introduction, therefore it is important to understand any genetic impact of ASF outbreaks on wild boar populations. The aim of this study was to assess how the propensity for an outbreak has shaped genetic variation in the wild boar population. A total of 491 wild boar samples were collected and genotyped using 16 STR markers. Allele richness varied between 15 and 51, and all SSR loci revealed a significant deviation from the Hardy–Weinberg equilibrium. Fixation indices indicated a significant reduction in heterozygosity within and between subpopulations. PCoA and STRUCTURE analysis demonstrated genetic differences between the western region which had had no outbreaks (restricted zone I) and the region with ASF infection (restricted zones II and III). It is concluded that environmental factors may play a particular role in shaping the regional gene flow and influence the genetic structure of the wild boar population in the region with ASF outbreaks.

## 1. Introduction

Wild boar (*Sus scrofa*), one of the most abundant ungulates in Europe, plays an important role in the ecosystem [1,2]. The increase in average temperatures and loss of snow cover due to a warming climate contribute to the wild boar population growth [3,4,5]. *Sus scrofa* is a species that has positive effects on natural communities [2]. At the same time, increasing densities and the expansion of wild boars can cause serious problems in agriculture and the transmission of diseases to livestock [6]. In 2007, the African swine fever virus (ASFV), a lethal haemorrhagic disease of wild boars, was introduced to Georgia, from where the virus continued its spread into Europe [7,8]. Since January 2014, ASF has spread to other European countries such as Estonia, Latvia, Lithuania and Poland, and has become endemic in wild boar populations. The first outbreak of ASF was detected in the eastern part of Lithuania near the border with Belarus, and since then the disease has spread to most of Lithuania [9]. The spread of ASFV epidemics has affected wild boar populations in Eurasia. According to Morelle et al. [10], the abundance of the wild boar population decreased by up to 85% in the first years of the ASF outbreak. To limit the spread of ASF, control measures such as sanitary culling induced mortality that could contribute to the wild boar population decline [11]. Many research studies have focused on genetic analysis to understand the phylogeographic [12,13,14,15,16] and population genetic structure of the European wild boar [17,18,19,20,21,22,23,24,25]. Although various studies of the wild boar’s genetic structure have been conducted, most of them have focused on the demographic history and investigations of the natural and artificial barriers. There is a lack of population genetic studies of how ASF outbreaks could contribute to a variation in the spatial genetic structure of *S. scrofa* populations.

This study aimed to investigate how the effects of the ASF outbreak may have affected the genetic structure of the wild boar population in Lithuania. 

## 2. Materials and Methods

### 2.1. Population Sampling and Genomic DNA Extraction 

**Figure 1:**
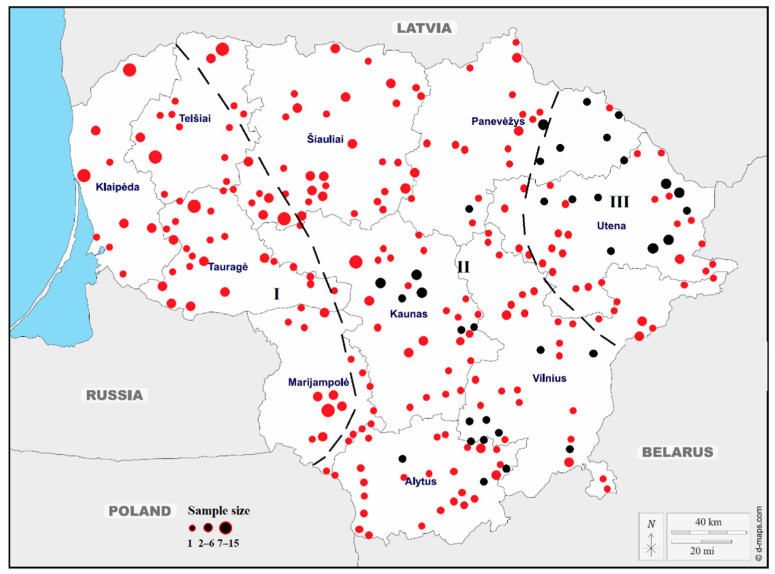
Geographic distribution of sampling sites across Lithuania. Black and red dots show collecting sampling sites of wild boars in 2014–2016 and 2017–2019, respectively. Black dashed lines indicate the restricted zones of African swine fever in Lithuania.

A total of 491 samples were collected from wild boars that had been hunted or killed on roads throughout the country over a five-year period (2014–2019). During the analysis, the spatial and temporal distributions of ASF in wild boars in Lithuania in the period from 2014 to 2018 were taken into account. The affected area was divided into restriction zones based on the Annex to Decision 2014/709/EU (https://eur-lex.europa.eu/legal-content/EN/TXT/?uri=CELEX%3A32014D0709, accessed on 5 May 2022) as follows: Part I (wild boar at risk, but no cases of disease; higher risk due to proximity to ASF infection), Part II (wild boar testing positive, but no spill over into domestic pig areas) and Part III (cases in wild boar and occasional spill over into domestic pig areas) (Figure 1). Blood samples were collected from hunted animals in the infected regions (listed in Parts II and III of the Commission Implementing Decision 2014/709/EU). Tissue samples and blood were also collected from wild boars hunted outside the infected regions or in the non-infected areas (listed in Part I of the Commission Implementing Decision 2014/709/EU). 

Wild boar samples were collected from various parts of Lithuania that have experienced varying rates of ASF spread. In some regions, wild boar subpopulations have experienced frequent and widespread outbreak events, while others in the least affected regions have had infrequent and local outbreaks. For each sample, the collection date and site were noted. The GPS coordinates of the sampling sites were used where possible. The number of samples, sampling period, and their sources of origin from all sampling sites are illustrated in Figure 1. All laboratory tests were performed at the National Food and Veterinary Risk Assessment Institute of Lithuania (NFVRAI). 

Blood samples were collected in vacutainer tubes that contained EDTA (0.5 mM, pH 8.0) and were stored at +4 °C until DNA extraction was performed. Tissue samples were preserved in 96% ethanol and stored at −20 °C. Total genomic DNA was isolated from blood and tissue samples using the DNeasy Blood and Tissue Kit (Qiagen, Valencia, CA, USA, Catalogue No. 69506) following the manufacturer’s instructions. The quantity and purity of isolated DNA were checked with a NanoDrop 2000 spectrophotometer (Thermo Scientific, Wilmington, DE, USA). Extracted DNA was stored at −20 °C until PCR amplification.

### 2.2. Microsatellite Analysis

All samples were genotyped for 16 microsatellite loci recommended by the International Society of Animal Genetics (ISAG)—Food and Agriculture Organization (FAO) [26]. DNA was amplified in 25 μL reaction mixes containing 1 μL of total DNA (about 30 μg), 12.5 μL of 2 × QuantiTect Multiplex PCR NoROX Master Mix (Qiagen, Hilden, Germany, Ref. 204743) and primers in 2 µM concentration for each. The cycling conditions were as follows: 10 min at 95 °C, followed by 30 or 35 cycles of 30 s at 95 °C depending on the used primer set, 30 s annealing at an optimal temperature ranging from 57 to 58 °C, 1 min at 72 °C, and finally 30 min at 72 °C. Fragment analysis was conducted via capillary electrophoresis on the PCR products generated from primer pairs labelled with different fluorescent dyes (FAM, HEX, and NED). Fluorescently labelled PCR products were separated on an ABI 3100 Genetic Analyzer (Applied Biosystems, Waltham, MA, USA), where a GeneScanTM-500 ROX size standard (Applied Biosystems, Waltham, MA, USA) was used as the internal standard. The length of DNA fragments was analysed manually in GeneMapper v. 4.0 (Applied Biosystems, Foster, CA, USA).

### 2.3. Data Analysis

Genetic diversity and polymorphism were analysed by locus and population. The genetic data analysed using Power Marker Software Version 3.25 were the major allele frequency (MAF), number of genotypes (N_G_), expected heterozygosity (He), heterozygosity (Ho), inbreeding coefficient measured as the deviation from random mating (Fis), and the polymorphism information content (PIC) [27]. The effective number of alleles (A_e_) and the number of private alleles (A_p_) were calculated using GenAleX 6.51b2 software [28]. Allelic richness (A_r_) was calculated using FSTAT, version 2.9.3.2 [29]. All departures from the Hardy–Weinberg equilibrium (HWE) were tested with a Markov chain algorithm (10,000 dememorisation steps, 100 batches and 1000 iterations) using Genepop v.4.0 [30]. The *p*-values were adjusted using a Bonferroni sequential correction for multiple comparisons, with an initial probability of *p* = 0.05 [31]. To estimate the presence of null alleles and genotyping errors, such as large allele dropout or stuttering in all sixteen loci, the software Microchecker V2.2.3 [32] and the Brookfield [33] null allele estimator with 1000 randomisations were used. 

The genetic structure of the wild boar population was assessed by means of the analysis of molecular variance (AMOVA), F-statistics, pairwise population comparisons, population differentiation and graphical representation using ARLEQUIN version 3.5.2.2 [34]. The statistical significance of AMOVA was tested by 10,000 permutations. Estimates of pairwise population differentiation (Fst) were evaluated between sampling sites based on the distance method, with the significance level set at *p* = 0.05. The exact test of population differentiation based on genotype frequencies was determined through 100,000 steps in a Markov chain and 10,000 dememorisation steps. GenAlEx was also used to perform a pairwise population matrix based on Nei’s genetic distance and to construct principal coordinates analysis (PCoA).

The population genetic structure was analysed with STRUCTURE 2.3.4 [35], using a model-based clustering algorithm that implements a Bayesian framework and the Markov chain Monte Carlo (MCMC) algorithm. To confirm the optimum number of subpopulations (K), ten independent runs for each value of K, ranging from 1 to 10, were conducted. Each run consisted of a burn-in period of 200,000 steps followed by 800,000 MCMC iterations. To determine the suitable number of clusters, the ad hoc statistic ΔK was calculated using the Evanno method [36] implemented in STRUCTURE HARVESTER, version 0.6.94 [37]. Mean posterior probability [LnP(D)] was also considered in order to determine the number of clusters within the dataset. A major clustering result at each K was visualised with CLUMPAK [38].

## 3. Results

### 3.1. SSR Genetic Diversity and Polymorphism

Among the studied sampling sites, two sampling sites (Alytus and Kaunas) had the highest A_r_ (12.638 and 12.347, respectively), and the sampling site at Klaipėda generated the lowest A_r_ (7.954) (Table 1). MAF varied from 0.283 (Kaunas) to 0.431 (Klaipėda), with a mean value of 0.347. The number of generated effective alleles (A_e_) ranged from 4.748 to 7.286, with a mean of 5.719. The highest A_e_ was observed at the Alytus sampling site, while the lowest A_e_ was from the Klaipėda sampling site. Among the private alleles, almost half (41.89 %) were found at two sampling sites (Vilnius and Panevėžys). Observed (Ho) and expected (He) heterozygosity values per sampling site varied from 0.568 (Utena) to 0.662 (Tauragė) and from 0.714 (Klaipėda) to 0.837 (Kaunas), respectively (Table 1). Highly significant deviations from Hardy–Weinberg after Bonferroni correction were exhibited at all sampling sites, mostly due to the deficit of heterozygotes. The inbreeding coefficient across sampling sites (Fis) was in the range of −0.129 to 0.320, with a mean of 0.217 (Table 1). A high level of inbreeding was found at the sampling site in Utena (0.320). All Fis values were determined to be positive. The mean Fis value of the population was 0.217, indicating that there was an excess of homozygous individuals in the sampling sites.

Results for individual microsatellite loci indicated that the total number of alleles per locus varied between 15 (sw72) and 51 (s0005). The value of allelic richness (A_r_) ranged from 7.619 (sw830) to 23.046 (s0005), with a mean of 12.230, He values ranged from 0.683 to 0.944 (average, 0.835), and Ho values from 0.405 to 0.798 (average, 0.623). The polymorphic information content (PIC) of the loci ranged from 0.664 in sw2410 to 0.942 in s0005, where the highest number of alleles per locus was observed in this study. Results showed that polymorphic information content (PIC) values were greater than 0.5 at every locus, which makes them useful in genetic diversity studies. The inbreeding coefficient across loci (Fis) ranged from 0.155 (s0005) to 0.408 (sw2410), with an average Fis of 0.255 (Table 2). Positive Fis was shown in thirteen loci, indicating excess homozygosity and the occurrence of deviations from HWE in these loci.

### 3.2. Null Alleles

Analysis using the MICROCHECKER software revealed that fourteen out of the sixteen loci scored had null alleles. The frequencies of null alleles per locus per population ranged from 0 to 0.264. It was identified that three loci (s0068, sw353, s0355) had potential null alleles at a high frequency (r > 0.2) in at least one subpopulation. These loci with null alleles were excluded from further analyses. The frequencies of the remaining microsatellite loci were below 0.20. 

### 3.3. Population Differentiation, Genetic Distance and Genetic Structure

Analysis of molecular variance (AMOVA) revealed that the variation among the sampling sites (Va) was 5.42%, the variation among individuals within locations (Vb) was 16.61%, and the variation within individuals was 77.97% (Table 3). The fixation indices had average values of overall F_st_, F_is_ and F_it_ of 0.054, 0.175 and 0.220, respectively. All three indices showed statistically highly significant differences (*p* < 0.001) from zero among and within all sampling sites, indicating that there was a loss of heterozygosity in the wild boar population and increased inbreeding among the subpopulations. 

**Figure 2:**
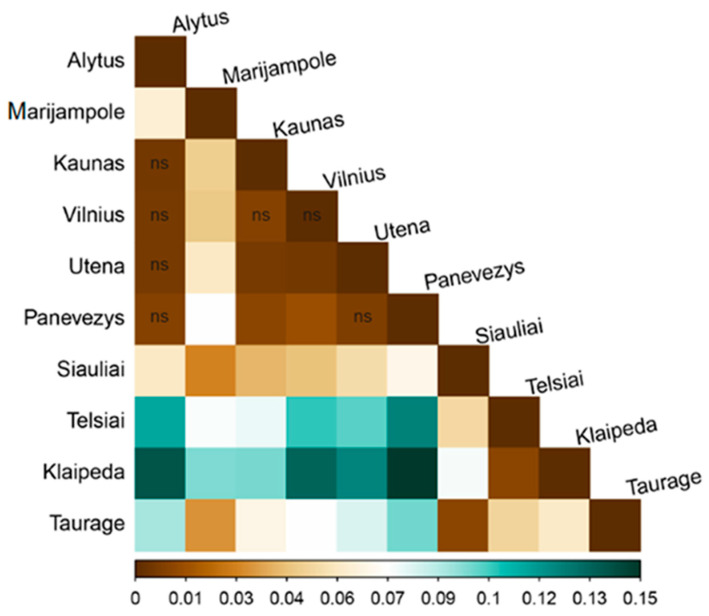
Pairwise Fst distances between studied wild boar sampling sites. The colour gradient represents the degree of genetic differentiation: low for Fst < 0.05, moderate for 0.05 < Fst < 0.15, high for 0.15 < Fst < 0.25 and very high for Fst > 0.25, according to the criteria for genetic differentiation of Wright (1978) (scale at the bottom of the figure (ns = not significant, blank significant *p* < 0.05, pairwise populations Fst value)).

Pairwise Fst values among sampling sites varied from 0.005 to 0.147, indicating mixed levels (from low to moderate) of genetic differentiation among the sampling sites (Figure 2, Supplementary Materials Table S1). The lowest pairwise Fst value (0.005) was observed between the Kaunas and Alytus, Utena and Alytus, and Utena and Vilnius sampling sites, while the highest value (0.147) was between the Panevėžys and Klaipėda sampling sites. Significantly higher (*p* < 0.05) genetic differentiation compared with all other sampling sites was observed at two sites: Telšiai and Klaipėda. Pairwise Fst values varied from 0.010 to 0.123 between the Telšiai sampling site and other sampling sites, and from 0.010 to 0.147 between the Klaipėda sampling site and other sampling sites. Several pairwise sampling sites with Fst < 0.05 indicated that the genetic differentiation between them was small and insignificant (Figure 2, Supplementary Materials Table S1).

The pairwise Nei’s (1983) genetic distances ranged from 0.071 to 0.768 between sampling sites (Supplementary Materials Table S1). The smallest genetic distance (0.062) was between the Telšiai and Klaipėda sampling sites, while the greatest genetic distance (0.768) was between the Klaipėda and Alytus sampling sites. 

**Figure 3:**
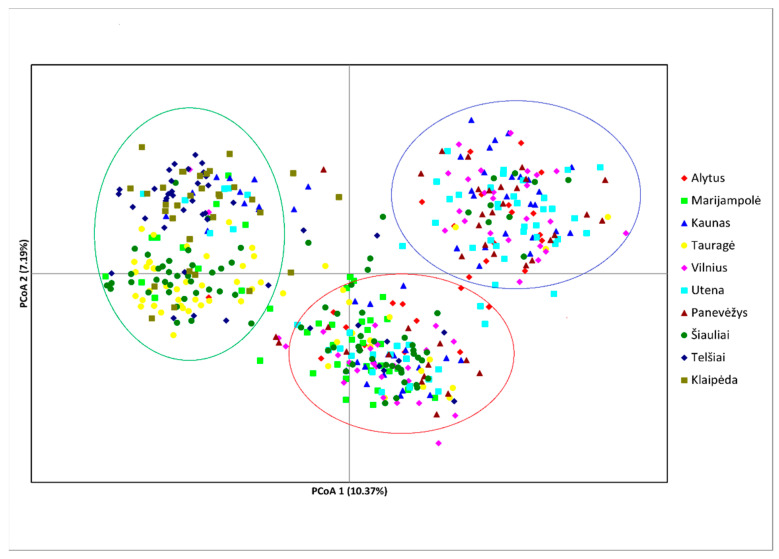
PCoA plot based on the genetic distance matrix of 491 wild boars collected from the sampling sites. Data obtained from analysis of 13 polymorphic microsatellite loci were used. A green ellipse indicates the grouping of almost all individuals from the western region, red and blue ellipses indicate that the remaining individuals from other regions tend to split into two mixed groups.

Principal coordinates analysis (PCoA) was performed to provide the genetic structure among all wild boar samples. The first three principal components explained 10.37%, 3.03% and 7.19% of the total variance, respectively (Figure 3). The PCA analysis revealed the arrangement of 491 wild boar individuals illustrated in the PCA plot, in which the three groups have a tendency to separate. A green ellipse indicates that the grouping mainly included the individuals from Tauragė, Klaipėda, Telšiai and a few individuals from the Šiauliai sampling site. However, a tendency for individuals from the western region to consist of two subclusters was also observed. The remaining individuals from the other sampling sites were split between the other two groups (red and blue ellipses) (Figure 3). Generally, one region-specific grouping, represented by the wild boar individuals in western Lithuania, was evident in the PCoA plot. Moreover, PCoA analysis separated individuals into the genetically differentiated group, which corresponded with the ASF-restricted area with no outbreaks.

**Figure 4:**
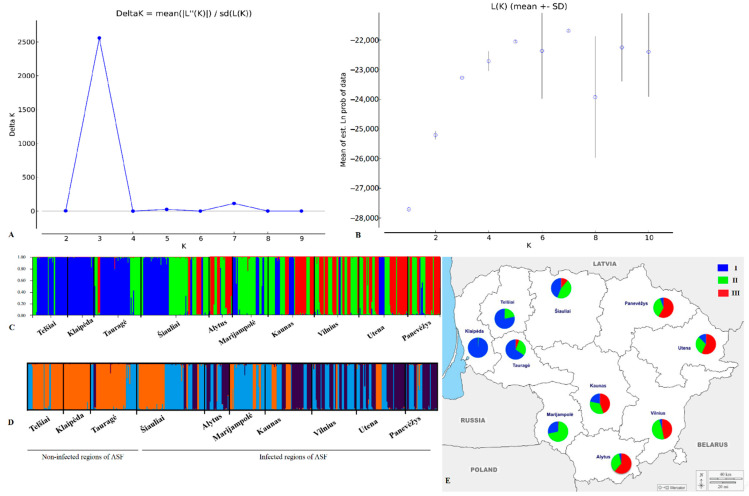
(**A**) Magnitude of delta K (ΔK) statistics for the *S. scrofa* collection based on 13 microsatellite loci. (**B**) STRUCTURE results: mean (±SD) of estimated Ln probability of the data for each K value. (**C**) Population structure of *S. scrofa* individuals collected from the sampling sites, estimated according to the Bayesian model by STRUCTURE program version 2.3.4 (K = 1–10). (**D**) Output from CLUMPAK, visualising major modes for K = 3 from the individual-based clustering performed in STRUCTURE. Each vertical line represents a single individual, and the colour shows the proportion of each individual assigned to each of the three genetic clusters. (**E**) Map showing the distribution of genetic groups determined by STRUCTURE analyses. Pie charts represent the proportions of each cluster.

In the structure analysis, LnP(D) and ΔK statistics were used to determine the most likely value of population genetic cluster K. As the LnP(D) increased from K = 1 to 5, the true number of genetic clusters (K) was difficult to determine. However, the ΔK statistic of Evanno et al. [36] detected the highest peak at K = 3 (Figure 4a,b). The distribution of genetic clusters at K = 3 showed a clear separation of the western part of the country, consisting of the Telšiai, Klaipėda and Tauragė sampling sites (Figure 4c,d). Individuals from the western region were mostly assigned to the first cluster (blue colour), whereas cluster 2 (green colour) and cluster 3 (red colour) were dominant in the remaining subpopulations (Figure 4c,e).

## 4. Discussion

More comprehensive sampling compared with a previous study undertaken and the high number of STR markers used produced 491 individual multilocus genotypes of wild boars across Lithuania. The analysis of genetic diversity and the population structure of wild boars was conducted with regard to the different prevalence estimates of ASF in affected areas of the country. This study aimed to extend the previous study [24] and obtain more insight into changes in the genetic structure of the wild boar population in Lithuania following the emergence of ASF. In the previous study, in which 15 microsatellites were analysed, no evidence was found of genetic differentiation among wild boars before the ASF outbreak in Lithuania [24].

In this study, the wild boar sample collection still retained a reasonable amount of genetic variation, with allele richness of 15 to 51 alleles per locus and an average PIC of 0.819. Genetic parameters such as allelic richness (Ar), number of effective alleles (Ae) and the polymorphic information content (PIC) had lower values at the three sampling sites (Telšiai, Klaipėda and Tauragė) in the western part of the country. Another measure of genetic variability is expected heterozygosity, with the highest value (0.837) found at the Kaunas sampling site and the lowest (0.714) at the Klaipėda sampling site. The values of this genetic diversity parameter were slightly higher than those determined in a recent study (He = 0.667), which showed the genetic structure of the wild boar population before the ASF outbreak [24]. However, the observed heterozygosity value (Ho = 0.623) was close to that quantified in the earlier study on wild boars (Ho = 0.627), despite the fact that the sample size in the current study was four times greater. Similar levels of Ho have also been detected in Bulgaria [18], Portugal [19], Croatia [39] and the Geneva region [25].

All SSR loci (100%) revealed a significant deviation from HWE after Bonferroni correction. This finding was consistent with previous studies [17,20,23,24,40], which have shown that deviations from HWE are relatively common in the wild boar population. The genetic imbalance in all microsatellite loci in the wild boar population may be associated with inbreeding in small local populations, null alleles, ecological events, a Wahlund effect or a reduction in the effective breeding population size (Ne) [33,41]. During the years of the epidemic (2014–2018), a significant decrease in the wild boar population was due to the death of the majority of animals from ASFV and the hunting of wild boar in ASF-affected regions [11]. The abundance of wild boar did not differ significantly in Lithuania until 2014 when, according to the monitoring data in 2014, the wild boar population exceeded 60,000 individuals. Since ASF arrived in Lithuania in 2014, the wild boar population has decreased to 22,000 individuals. According to data from the Ministry of Environment of the Republic of Lithuania, during the period 2014–2018, 223,184 wild boars were hunted, with the number of hunted animals decreasing from 46,462 in 2013–2014 to 18,016 in 2018–2019 (https://am.lrv.lt, accessed on 5 May 2022). The previous analysis indicated that in Lithuania the number of ASF-positive wild boar carcasses ranged from 20.2% in 2014 to 79.7% in 2017 [42]. Selective hunting of female wild boars was an EU control strategy for ASF [43]. Since 2015, the promotion of adult and subadult female boar hunting is used as a means of reducing the boar population [11]. As previously discussed, selective removal of wild populations can influence the mating structure, leading to genetic changes [44]. Small effective population sizes can lead to a decline in heterozygosity and the loss of rare alleles [45]. 

The presence of null alleles might reduce the genetic diversity within populations and cause an overestimation of population differentiation [46]. In this study, three pairs of primers with greater frequencies of null alleles were identified and discarded from further analysis. Furthermore, the presence of null alleles at relatively low frequencies has no significant effect on the results of genetic analysis [47].

In the present study, the fixation indices Fst, Fis and Fit indicated a significant reduction in heterozygosity within and between subpopulations. Fis values significantly higher than zero indicate more inbreeding than would be expected at random [48]. As reflected in the AMOVA, genetic differentiation was low in the overall wild boar collection (Fst = 0.054), as evidenced by the highly significant level (*p* < 0.001) of inbreeding among individuals within the population (Fis = 0.175). Similar findings have also been reported for the same species in the earlier study conducted before the ASF outbreak in Lithuania [24].

With regard to all pairwise differences (linearised Fst) in this study, the distribution of Fst represented moderate genetic divergence (0.000 < Fst < 0.147) between sampling sites in general. Fst was observed to be small between sampling sites in western Lithuania and had a greater differentiation from the rest of the sampling sites. From a genetic viewpoint, population genetic differentiation can arise from migration, geographical barriers, genetic drift and gene mutation [49]. A recent study has shown that habitat fragmentation makes a small contribution to genetic differentiation in the wild boar population in Lithuania [24]. One possible explanation for this could be the spatial distribution of ASFV, with the disease spreading westwards through the wild boar population; however, a few municipalities in the very western part of Lithuania are not affected by ASF. It is possible that outbreak events of ASF also have an impact on the wild boar’s genetic structure. 

In this study, the analysis with the STRUCTURE program showed that the population of wild boar in Lithuania is grouped into three major lineages when K = 3 (Figure 4a,b). The results obtained supported the Fst, PCoA and genetic distance results. Individuals in the western region were identified as having similar genetic compositions (green and blue colours predominated), whereas the regions affected by ASF were characterised by a similar genetic structure (green and red colours predominated). The possibility cannot be ruled out that the ASF epidemic may have affected the genetic diversity of wild boars in the sampling sites affected by ASF. The genetic structure in the western region where no cases of the disease have been reported may have remained unchanged. According to a previous study conducted in Lithuania before ASF, the population of wild boar exhibited a lack of genetic structure [24]. The information about the existence of three genetic groups is consistent with the findings from earlier studies [19,20,21]. In contrast, one genetic cluster was confirmed in the Lithuanian wild boar population before ASF [24] and two genetic groups were identified in wild boar populations in the Carpathian Basin [23]. The homogenous genetic structure was also confirmed in a recent study that showed the clustering of all individuals into a single genetic group in three areas of the Geneva region connected by an ecological corridor [25]. The contrasting findings in the present study compared with previous studies may be related to the geographic scale of the study, the use of different markers and the sample size. Furthermore, a study that analysed mtDNA D-loop sequences of wild boar in central and eastern Europe identified three mtDNA clades [16]. A possible explanation for the existence of three genetic groups may be associated with various factors such as multiple climatic fluctuations and past demographic and migratory events, and human-mediated translocations can affect the current patterns of the wild boar population’s genetic structure [20,23,50,51].

## 5. Conclusions

This study assessed the genetic diversity and population structure of wild boars following the emergence of ASF using microsatellite markers. To examine changes in the population’s genetic structure, the findings were compared with the genetic structure of the wild boar population studied before the ASF outbreak. Sampling sites in the western part of Lithuania (a region with no ASF infections) were found to be genetically distinct from other parts of the country (regions infected with ASF), while in the earlier study, no differentiation between sampling sites was found. These findings suggest that environmental factors play a particular role in shaping regional gene flow and influence the genetic structure of Lithuania’s wild boar population.

## Figures and Tables

**Table 1:** Microsatellite diversity and polymorphism of wild boar by sampling site.

Sampling Sites	N	A_r_	MAF	A_e_	N_G_	A_p_	He	Ho	Fis	PIC	HWE *p*-Value
Telšiai	43	9.377	0.394	5.047	17.308	7	0.742	0.601	0.201	0.718	0.000 **
Klaipėda	32	7.954	0.431	4.194	13.000	4	0.714	0.625	0.140	0.685	0.000 **
Tauragė	56	9.392	0.397	4.630	18.615	1	0.752	0.662	0.129	0.725	0.000 **
Marijampolė	43	9.191	0.389	4.748	16.230	2	0.749	0.647	0.162	0.721	0.000 **
Alytus	28	12.638	0.293	7.188	15.615	7	0.823	0.654	0.224	0.805	0.000 **
Kaunas	56	12.347	0.283	7.286	24.154	4	0.837	0.633	0.252	0.820	0.000 **
Vilnius	53	11.985	0.321	6.370	22.385	17	0.816	0.620	0.250	0.799	0.000 **
Šiauliai	82	10.176	0.337	5.566	24.692	9	0.795	0.644	0.195	0.773	0.000 **
Utena	59	11.902	0.297	6.399	24.077	9	0.826	0.568	0.320	0.809	0.000 **
Panevėžys	39	11.711	0.331	5.757	18.000	14	0.807	0.578	0.296	0.790	0.000 **
Mean	-	10.513	0.347	5.719	19.408	6.7	0.786	0.623	0.217	0.765	0.000 **

N—sample size, A_r_—allelic richness, MAF—major allele frequency, A_e_—number of effective alleles, N_G_—number of genotypes, A_p_—private alleles, He—expected heterozygosity, Ho—observed heterozygosity, Fis—inbreeding coefficient, PIC—polymorphism information content, HWE *p*-value—exact test for HWE using a Markov chain for all loci (significant, **: *p* < 0.01).

**Table 2:** Microsatellite diversity and polymorphism by locus.

SSR Marker	MAF	N_A_	N_G_	A_r_	He	Ho	PIC	Fis	HWE *p*-Value	Nm
sw24	0.310	20	67	11.070	0.822	0.688	0.802	0.164	0.000 **	4.334
s0107	0.210	29	102	14.646	0.895	0.587	0.887	0.346	0.000 **	3.836
s0386	0.233	19	60	12.795	0.892	0.684	0.883	0.234	0.000 **	4.551
sw72	0.388	15	31	7.918	0.774	0.538	0.748	0.306	0.000 **	8.825
tnfb	0.177	33	83	16.340	0.912	0.748	0.906	0.182	0.000 **	7.152
s0070	0.276	29	59	11.825	0.858	0.646	0.845	0.249	0.000 **	4.221
s0026	0.333	17	40	10.093	0.817	0.670	0.798	0.181	0.000 **	3.366
s0155	0.327	20	37	9.885	0.809	0.495	0.788	0.389	0.000 **	1.705
s0005	0.147	51	179	23.046	0.944	0.798	0.942	0.155	0.000 **	6.032
sw2410	0.534	26	46	10.243	0.683	0.405	0.664	0.408	0.000 **	2.747
sw830	0.418	16	30	7.619	0.713	0.450	0.671	0.370	0.000 **	2.245
sw632	0.163	20	72	14.024	0.909	0.729	0.902	0.199	0.000 **	3.912
swr1941	0.325	19	46	9.490	0.826	0.659	0.808	0.202	0.000 **	4.761
Mean	0.295	24.153	65.53	12.230	0.835	0.623	0.819	0.255		4.437

SSR—simple sequence repeat or microsatellite, MAF—major allele frequency, N_A_—number of alleles, N_G_—number of genotypes, A_r_—number of alleles per genotype, allelic richness, He—genetic diversity, Ho—observed heterozygosity, PIC—polymorphism information content, Fis—inbreeding coefficient, HWE *p*-value—exact test for HWE using a Markov chain for all loci (significant, **: *p* < 0.01), Nm—estimates of gene flow.

**Table 3:** Analysis of molecular variance (AMOVA) results for sampling sites of wild boar based on various genetic groupings.

Source of Variation	d.f.	Sum of Squares	Variance Components	Percentage of Variation	F-Statistics	Value	*p*-Value
Among sampling sites	9	253.061	0.23884 Va	5.42	Fst	0.054	*p* < 0.001
Among individuals within sampling sites	481	2358.147	0.73236 Vb	16.61	Fis	0.175	*p* < 0.001
Within individuals	491	1688.000	3.43788 Vc	77.97	Fit	0.220	*p* < 0.001
Total	981	4299.208	4.40907	100.00			

## Data Availability

The data presented in this study are available on request from the corresponding author.

## Supplementary Materials

The following supporting information can be downloaded at: https://www.mdpi.com/article/10.3390/genes13091561/s1, Table S1: Pairwise Nei’s genetic distance coefficient (above diagonal) and pairwise FST values (below the diagonal) for the studied wild boar subpopulations.
