# Supplementary material for: Changes in the Genetic Structure of Lithuania’s Wild Boar (Sus scrofa) Population Following the Outbreak of African Swine Fever
Source: Genes (Basel). 2022 Aug 30;13(9):1561. doi: 10.3390/genes13091561 (PMC9498859; doi:10.3390/genes13091561)
Supplement: Supplementary file 1 [file genes-13-01561-s001.zip › genes-1768589-supplementary.pdf]

Table S1. Pairwise Nei's genetic distance coefficient (above diagonal) and pairwise FST values (below the diagonal) for the studied wild boar from different sampling sites.

	**Alytus**	**Marijampolė**	**Kaunas**	**Vilnius**	**Utena**	**Panevėžys**	**Šiauliai**	**Telšiai**	**Klaipėda**	**Tauragė**
**Alytus**	-	0.321	0.103	0.093	0.099	0.105	0.316	0.592	0.768	0.463
**Marijampolė**	0.062*	-	0.238	0.212	0.273	0.340	0.123	0.294	0.398	0.131
**Kaunas**	0.005	0.048*	-	0.085	0.079	0.109	0.221	0.350	0.438	0.315
**Vilnius**	0.006	0.046*	0.009*	-	0.065	0.105	0.215	0.489	0.654	0.316
**Utena**	0.005	0.059*	0.006	0.005	-	0.077	0.266	0.468	0.598	0.370
**Panevėžys**	0.009	0.073*	0.010*	0.014*	0.007	-	0.320	0.593	0.766	0.454
**Šiauliai**	0.059	0.029*	0.041*	0.044*	0.054*	0.067*	-	0.213	0.313	0.058
**Telšiai**	0.113*	0.074*	0.077*	0.105*	0.100*	0.123*	0.052*	-	0.071	0.205
**Klaipėda**	0.137*	0.096*	0.097*	0.133*	0.123*	0.147*	0.075*	0.010*	-	0.258
**Tauragė**	0.091*	0.033*	0.067*	0.072*	0.081*	0.098*	0.010*	0.051*	0.059*	-

*Indicates a significant difference at $p < 0.05$ level.
